# Understanding immunity: an alternative framework beyond defense and strength

**DOI:** 10.1007/s10539-023-09893-2

**Published:** 2023-02-15

**Authors:** Martin Zach, Gregor P. Greslehner

**Affiliations:** 1 Department of Analytic Philosophy, Institute of Philosophy, Czech Academy of Sciences, Jilská 352/1, 110 00 Prague, Czech Republic; 2 Department of Philosophy, University of Vienna, Universitätsstraße 7, 1010 Vienna, Austria

**Keywords:** Immune system, Contextuality, Regulation, Trade-offs, Strong immunity, COVID-19, SARS-CoV-2

## Abstract

In this paper we address the issue of how to think about immunity. Many immunological writings suggest a straightforward option: the view that the immune system is primarily a system of defense, which naturally invites the talk of *strong* immunity and *strong* immune response. Despite their undisputable positive role in immunology, such metaphors can also pose a risk of establishing a narrow perspective, omitting from consideration phenomena that do not neatly fit those powerful metaphors. Building on this analysis, we argue two things. First, we argue that the immune system is involved not only in defense. Second, by disentangling various possible meanings of ‘strength’ and ‘weakness’ in immunology, we also argue that such a construal of immunity generally contributes to the distortion of the overall picture of what the immune system is, what it does, and why it sometimes fails. Instead, we propose to understand the nature of the immune system in terms of contextuality, regulation, and trade-offs. We suggest that our approach provides lessons for a general understanding of the organizing principles of the immune system in health and disease. For all this to work, we discuss a wide range of immunological phenomena.

## Introduction

Philosophy of immunology has grown into a small field within philosophy of science (see Pradeu 2019; Swiatczak and Tauber 2020).^1^ Here we contribute to the existing scholarship and consider a general framework (or account) of immunity. Our use of the terms ‘framework’ or ‘account’ indicates that we do not mean to propose a new *theory* of immunity (although there is a need for such a ‘general theory of immunity’, see, e.g., Eberl and Pradeu 2018). Instead, we want to address the widespread *mindset* from which one views the immune system, and we ultimately propose an alternative framework which better reflects recent advances.

The dominant characterization of the immune system found in immunological literature is that of a defense system which is perceived in terms of ‘strength’ and ‘weakness’ and which engages in ‘strong’ or ‘weak’ responses. War metaphors have always been very popular in immunology, framing immune phenomena in terms of fortresses, armies, and battles. Generally, while metaphors play an essential role by helping in the conceptualization of phenomena and the organization of knowledge, they may also shape a narrow perspective, overlooking other important aspects. Therefore, we first scrutinize the mindset that the immune system is a defense system. Second, we ask whether and how the strong/weak framework could shed light on the immune system and the immune response. We conclude that the strong/weak defense framework biases the understanding of the general nature of the immune system because it neglects immune activities that are unrelated to defense and that do not fit the strong/weak narrative. As a result, we argue that the view of the immune system as a strong/weak defense system should be reconsidered.

After this critical assessment, we propose another framework which we harvest from the recent immunological literature—one that, once made explicit, provides insight into the general organizing principles of immunity. The framework we suggest as an alternative way to think about immunity provides core tools for framing, understanding, and studying the immune system. It emphasizes the crucial aspects of *co*ntextuality and *re*gulation of immunity, and the biological *t*rade-*o*ffs (CORETO) which the immune system exhibits. Although these concepts are well-known to immunologists, they usually appear as descriptors of discovered states of affairs. By explicitly analyzing these concepts, we propose that they should play a more prominent role in thinking about immunity, and that such a framework is a viable alternative to the problematic view of immunity as strong/weak defense.

Finally, while we expect specialists to be in agreement with our exposition of empirical data, our conceptual analysis and the claims we make are not immediately self-evident, but took time to uncover and develop. We submit that putting the individual pieces regarding immunity together, along the lines we suggest, may help to make the right distinctions and connections when trying to understand immunity.

## On the idea of immunity and its function

Before moving on, let’s make some terminological decisions. First, for our purposes here, we use ‘immunity’ interchangeably with ‘immune system’.^2^ Second, as customary we distinguish between immunity/immune system as something that is the state of the whole system and a particular immune response, which can be characterized in terms of intensity, particular constituents involved in the response, whether it is local or systemic, and the onset and duration of the response, etc.

### How infections and defense have shaped the image of immunity

The immune system is generally conceptualized as a defense system which protects the organism from pathogens. Indeed, “[t]he origins of immunology centered on an animal host suffering pathogenic invasion. […] those that lived mounted an effective immune defense” (Tauber 2017, p. 23). Even now many suggest that defense is the immune system’s only function—that we “have an immune system for one reason and one reason only”, which is to prevent microbes from overrunning us (Clarke 2007, p. 3). Broadly, we can identify two lines of thought responsible for the predominance of the defense perspective: clinical and evolutionary reasons.

As some scholars of immunology have noted, such a view may owe its prevalence to a large extent to early the success of vaccines, the deep historical roots of the germ theory of disease, and the research following the tradition of Pasteur and Koch in which the immune system’s purpose is to protect the host by destroying pathogens (Eberl 2016). It is easy to see how this has shaped the perspective of physicians and the ensuing biomedical research, with the aim to understand and mitigate the impact of harmful microbes.

There is also an evolutionary story framed as an arms race between pathogens and host, where the host would develop increasingly complex defense mechanisms, and the pathogens, in turn, increasingly sophisticated ways to evade the immune system’s defenses (Clarke 2007; Crawford 2018).

Relatedly, what continues to shape how many scientists and physicians think and write about the immune system is the general conceptualization of the immune system centered around the idea that it distinguishes between ‘self’ and ‘non-self’ (Burnet 1969): the immune system learns to tolerate ‘self’, and when it recognizes ‘non-self’ (such as pathogens) it triggers a response against it, thereby defending the ‘self’.

Thinking about the immune system in terms of a defense system naturally invokes other, related intuitions such as the talk of *strong* immunity and *strong* immune response (see Martin 1994 for a critical analysis). In Tauber’s words, “[t]he strength of the ‘host defense’ orientation resides in a long and prominent clinical history” (Tauber 2017, p. 2).

Connecting the dots, we may also find passages such as the following one:

“The evolutionary advantages of a strong defence system are obvious in protecting against pathogens, and as a strong immune response is dependent on energy sources, one can also argue that the integration of these systems and their cooperation in responding to fluctuations in the energy and nutrient environment would be highly advantageous” (Hotamisligil 2017, p. 177).

### The role of metaphors in immunology

Metaphors abound in immunology. Arguably the most dominant view centers around war metaphors, conceiving of the immune system in terms of a fortress, an army of cells, and an arsenal of weapons defending the host, attacking and killing foreign invaders. As noted above, one would like these defensive measures as strong as possible (see Martin 1994 for a critical analysis and vivid illustration). Such use of war metaphors has been deeply rooted in immunology throughout its history, not just as communication devices with a wider audience, but shaping the very way scientists think, understand, and build theories in immunology (Martin 1994; Tauber 1994; Löwy 1996; Institute of Medicine 2006).

The claim that science is soaked in metaphorical language is scarcely contested. Rather, “in science, metaphor is widely considered an essential tool for understanding” (Ball 2011). Metaphorical language is often used to understand and communicate complex phenomena that are not completely understood by referring to other, more familiar concepts: “*The essence of metaphor is understanding and experiencing one kind of thing in terms of another*” (Lakoff and Johnson 1980, p. 5, original italics). Using metaphors helped shape the understanding of phenomena through the lens of something familiar, allowing to navigate and organize what otherwise would constitute a complex mess of individual facts. By focusing on certain aspects, one will also see and conceptualize along these lines, i.e., “through a certain lens” (Reynolds 2018).

The way metaphors are shaping scientific endeavors have received ample scholarly attention (Black 1962; Fox Keller 1995; Reynolds 2022). It has been proposed that, in science, metaphors serve at least three functions which are often interrelated in various ways (Bradie 1999; see also Kampourakis 2020). Metaphors have a *heuristic function* which helps scientists explore new phenomena by referring to other, already understood phenomena. Such a function is also achieved by drawing on a variety of analogies, a practice which has been documented in empirical studies of how immunologists reason (Dunbar 2002).

Additionally, metaphors have a *rhetorical function*: they play an important role in science education and communication. More importantly, rather than being merely instruments of getting a point across to a larger audience, “metaphors have profound influences on how we conceptualize and act with respect to important societal issues” (Thibodeau and Boroditsky 2011).

Finally, metaphors also have an indispensable *theoretical function*, i.e., they facilitate the understanding and explanation of phenomena. For instance, the self/non-self framework was put forth as an explanation of the basis of immune response (Tauber 1994). Notwithstanding their undisputable usefulness, it has also been well recognized that metaphors can also obscure understanding and lead one astray. As Philip Ball has put it:

“Books of life, junk DNA, DNA barcodes: all these images can and have distorted the picture, not least because scientists themselves sometimes forget that they are metaphors. And when the science moves on—when we discover that the genome is nothing like a book or blueprint—the metaphors tend, nonetheless, to stick. The more vivid the image, the more dangerously seductive and resistant to change it is” (Ball 2011).

Another prominent example concerns the dominant conception of the organism as a machine, scrutinized by Nicholson (2014). Similarly, in immunology powerful metaphors have served as the lens through which immunological phenomena have been studied, providing a much-needed structure to known immunological observations. The most prevalent among these metaphors are the self/non-self framework and the idea of the immune system as a defense system that is somehow characterized in terms of strength or weakness. However, as hinted above, these metaphors can be so seductive that one is at risk of forgetting the fact that they serve merely as lenses. Looking through them long enough may result in omitting from consideration phenomena that do not neatly fit those powerful metaphors. More poetically put, if all we have is a hammer, everything looks like a nail.

### What could ‘strength’ and ‘weaknesses’ mean?

Notions such as ‘strong (or weak) immune response’ and ‘strong (or weak) immunity’ frequently appear in immunological writings. However, although intuitive, these notions can take on a variety of meanings. In what follows we provide a non-exhaustive categorization of several options of how one can understand these notions (and their limitations).

We submit that the most prominent sense in which these terms are employed in the immunological literature relates to a qualitative assessment of some underlying quantitative measurement. For its attractiveness and convenience, we call this prevalent usage the ‘quantitative allure’.

(i) *Quantitative allure*. As noted above, these notions are often used as an assessment of a quantitative measurement of certain immune components and features: e.g., assays allowing measurements of cytokine production, the number of cells, the titers of (neutralizing) antibodies as a proxy for protective immunity, binding affinities, and so on. For example, in a study by Long et al. (2020), the authors found differences in the levels of specific antibodies, on the basis of which they stated that “data suggest that asymptomatic individuals had a weaker immune response to SARS-CoV-2 infection [than symptomatic individuals]” (Long et al. 2020, p. 1200). These notions are also used with regards to various epidemiological measurements. For instance, high percentages of vaccine efficacy of the COVID-19 vaccines, which express the relative risk reduction, are interpreted as conferring ‘strong protection’. Although suggestive, closer inspection reveals cracks in the interpretation of measurements within the strong/weak framework.

Importantly, while a combination of certain quantitative measurements can sometimes be straightforwardly linked to functional outcomes, in many cases such quantitative measurements *alone* may not be very informative.^3^ In the course of an immune response, two individuals may reach the same levels of the measured quantity but at different rates, resulting in significantly different outcomes: for example, a study by Lucas et al. (2020a) found that while deceased patients produced a specific antibody response against SARS-CoV-2 comparable to survivors, such a response was delayed. Thus, the *temporal aspect*, i.e., kinetics, must not be overlooked: one of the factors that correlates with (viral) control is the time-dependent production of antibodies rather than the specific antibody levels per se.

Similar problems arise for other measurements. Consider binding affinities—trying to straightforwardly link molecular binding (recognition) to a specific immune response is known to be problematic. Binding affinities can be quantitatively measured and are often interpreted as ‘strong’ and ‘weak’, respectively. However, the functional response depends on a lot of additional factors. For example, various thresholds at different regions of the spectrum of binding affinities can lead to non-responsiveness, deletion, and alternative developmental programs of corresponding cell types; too ‘strong’ and too ‘weak’ affinity can also have the same functional outcome. Similarly, it is well known that the functional outcome of recognition (an individual binding event) is dependent on the integration of multiple signals and the local environment.

Furthermore, the *qualitative* modifications such as the different sialylation patterns rather than simply the sheer quantities play an important role: for example, patients suffering from severe COVID-19 have increased levels of afucosylated antibodies (i.e., IgGs lacking fucose) compared to patients with mild symptoms (Larsen et al. 2021). Interestingly, while afucosylated IgGs exacerbate COVID-19, they appear desirable in the context of HIV infection (Larsen et al. 2021).

Finally, a drop in a quantity should not be regarded as a drop in ‘strength’. For example, the decline of antibodies after infection or inoculation is to be expected and physiologically required. Yet, the decline need not be associated with the weakening of immunity. Let us explain. One may, indeed, become more susceptible to an infection, following the decline of neutralizing antibodies. However, owing to a previous encounter or a vaccine, the individual should be equipped with memory responses, thus in an immunologically *different state*.^4^

The point here is not to suggest that immunologists are unaware of these empirical facts (they obviously are), but rather that the simplistic idea of operationally defining strength and weakness in terms of a quantitative measurement, albeit somewhat convenient, does little to improve immunological statements. Rather than relying on such language, there are other, perhaps more adequate descriptive terms, such as ‘elevated’, ‘increased’, ‘high’, ‘augmented’, and ‘dampened’, ‘decreased’, ‘low’, ‘waning’, etc., which might be used in similar (perhaps less problematic) ways. Such terms seem less laden with additional expectations and do not lend themselves to unwarranted functional or evaluative interpretations, as opposed to speaking of ‘strong/weak immune response’.

Along these lines we can see that the strong/weak framework may seem appealing for denotating desirable and undesirable states:

(ii) *Normative connotation*. ‘Strong defense’, ‘boosting’ immunity, or a ‘strong immune response’ may be viewed as desirable. Perhaps the most common example is the use of vaccines, which are in fact highly desirable. Still, the connection with these metaphors is spurious and generally does not really work all that well. In many cases, ‘strong’ response may lead to pathology or come at a cost. For example, it is well established that an overly activated immune system can result in lots of tissue damage, or even the life-threatening condition called cytokine release syndrome. Or consider the question of tissue repair: a ‘strong immunity’ in that case would amount to excessive tissue repair, giving rise to fibrosis (Medzhitov et al. 2012). Similarly, stimulating or ‘boosting’ an immune system by using immunomodulatory substances is hardly ever a straightforward matter as evidenced by, for instance, the use of checkpoint inhibitors in cancer immunotherapies which allow certain cells to overcome their normal physiological limits but may also give rise to autoimmune diseases (Ribas and Wolchok 2018).

Furthermore, the consideration of several other immunological phenomena suggests additional issues for the strong/weak framework, making it appear even less attractive.

(iii) *Paradoxical connotation*. Immunodeficiency^5^ invites the intuition that the issue is one of a ‘weak’ immunity or response. However, the same immunodeficiency could also concern an issue of a ‘strong’ immunity or response and it is not clear which notion should apply. Consider, for example, an autoimmune disease called IPEX (immune dysregulation, polyendocrinopathy, enteropathy, X-linked), which develops as a result of a mutation in the gene encoding the transcription factor FoxP3 which plays a prominent role in the development and functioning of regulatory T cells. In this disease, the crucial suppressive function of regulatory T cells is impaired. Consequently, the affected individual suffers from a host of conditions including lymphoproliferation, thyroiditis, insulin-dependent diabetes mellitus, enteropathy, and other immune disorders (Rich et al. 2019). Thus, although an immunodeficiency does indeed refer to a defect, that defect may result in an unwanted response that is ‘too strong’, i.e., one that is not kept in check. Or consider the case of functional autoantibodies which may give rise to an autoimmune disease in which the immune system responds ‘too strongly’ against ‘self’ antigens, breaking the mechanisms of immunological tolerance. However, the outcome of such a response may result in a defective—perhaps ‘too weak’—response in another context. For instance, autoantibodies targeting neutrophils cause neutropenia, i.e., the depletion of neutrophils, which leaves the individual particularly susceptible to infection by pyogenic bacteria; one of the therapeutic approaches is the removal of the spleen, which plays a major role in clearances. So, applying either notion of strong or weak seems to be an arbitrary matter.

(iv) *Not applicable because not amenable to change*. Thinking in terms of a continuum between strong and weak immunity is sometimes invalid. For example, a thymectomy or splenectomy, i.e., the removal of the thymus or the spleen, respectively, impairs some of the functions of the immune system. More specifically, a neonatal thymectomy prevents the development of mature T cells whereas a thymectomy in adulthood has little impact, since the pool of naïve T cells forms early on, and the thymus deteriorates with age, beginning soon after puberty (Murphy and Weaver 2017). Perhaps less dramatically, the removal of the spleen confers a lifelong susceptibility to devastating infection by encapsulated bacteria such as *Streptococcus pneumoniae*, which requires that the affected individuals take antibiotics prophylactically and are vaccinated against pneumococcal infection. Individuals without a functional spleen lack the mononuclear phagocytes normally found within the spleen which clear this organism from the blood (Murphy and Weaver 2017). However, such individuals are fully competent in launching a response against many other pathogens including many viruses, just like immunocompetent individuals. Clearly, then, as the examples of neonatal thymectomy and splenectomy illustrate, it makes little sense to think in terms of there being a scale on which one may move between weakness and strength: it is simply either there or missing.

Other times, it may be intuitive to think in terms of such a continuum between strong and weak, since, for instance, nutrition can be progressively improved in some sense. Malnutrition is known to affect cell-mediated immunity and is a major risk factor for many infectious diseases. Thus, intuitively we would believe that the better the nutrition the ‘stronger’ the immune protective effect. However, in contrast to this general belief, in certain specific circumstances deficiency may actually confer some additional level of protection. In particular, iron, though required in many immune-related pathways, happens to be essential for many bacteria, fungi and protozoa: it turns out that a certain degree of iron deficiency defined using ferritin and transferrin saturation in African children reduces the growth rate of the causative agent of malaria (Muriuki et al. 2019). Thus, even the assumption that a good diet is always associated with increased protection turns out to be wrong in at least some, albeit very specific, cases (compare also (iii)).

(v) *Lack of meaning conveyed*. Many phenomena and functions of immunity cannot be meaningfully captured by these notions altogether. Even when it is not a question of all-or-nothing, it often makes no sense to speak of a certain phenomenon or trait to be ‘stronger’ or ‘weaker’ at all. For instance, the concept of strong immunity would convey no meaning when applied to homeostasis. Other dynamical states like health or processes like development, in which the immune system plays an important role cannot be meaningfully called strong or weak either. While perhaps displaying certain robustness features, they do not fall under the conventional meanings implied.

(vi) *Mischaracterizing the immune system in interaction with other (physiological) systems.* Framing the immune system’s contribution to other systems in terms of ‘strong’ and ‘weak’ would be confusing rather than helpful in understanding the immune system’s role in interaction with those systems—like the nervous system, where the immune system participates in regulating behavior and cognition. Overall, there might be even more cross-talk and functional similarity between the nervous system and immune system in regulating and balancing other physiological systems in response to internal and external contexts (Dantzer 2018). In a similar way, it would be inadequate to refer to the central nervous system—or cognition—as ‘strong’ or ‘weak’.

Taken together, (i)–(vi) discussed above illustrate three important issues. First, the strong/weak framework gives the false impression that the immune system can be described along this (one) dimension. Second, while these notions rest on some intuitive understanding, they in fact prove to be elusive. Additionally, to an uninitiated reader the different usage of the terms may appear confusing. Recall the quotes from above. While suggesting a role for *‘strong immune response*’, Hotamisligil (2017, p. 177 italics added) states that “*the evolutionary advantages of a strong defence system are obvious in protecting against pathogens.”* Colloquially, it is also often stated that vaccines provide strong protection against disease which is achieved by a strong immune response realized by the induction of high antibody titers. In contrast, Long et al. (2020, p. 1200) report differences in the levels of infection-induced antibodies, claiming that “data suggest that asymptomatic individuals had a weaker immune response to SARS-CoV-2 infection [than symptomatic individuals]”, thereby suggesting a weak immune response is advantageous in protecting against the SARS-CoV-2 pathogen. Rather than presenting conflicting empirical claims, such statements in which the authors draw on the strong/weak framework suggest that the authors are talking at cross-purposes, illustrating that these notions are generally not well-defined. Third, and crucially, on the one hand we acknowledge it is hard to question the intuitive appeal of the notions of strong and weak, and their apparent utility in discussing immune phenomena. On the other hand, however, the explicit and systematic analysis of the variety of meanings of these notions provide reasons to at least give pause to anyone who is accustomed to and invested in using them. Overall, while in (i) and (ii) we reveal problems with the prevalent usage in immunology, (iii)–(vi) constitute additional challenges to adopting the strong/weak framework.

### Immunity is not just defense

Despite its intuitive appeal and rich history, both the self/non-self framework and the idea of defense have to be called into question. Some well-known problems have already been discussed in the scientific and philosophical literature (see, e.g., Pradeu 2012; Tauber 2017). Among the most notable issues, we find that the immune system is responsible for and involved in many other activities than *defense* against ‘foreign’ (i.e., ‘non-self’) material, including tissue repair, the clearance of damaged or dead cells and debris (i.e., ‘self’), developmental processes, the maintenance of homeostasis, and many more (Swiatczak 2013; Laurent et al. 2017; Rankin and Artis 2018; Pradeu 2019). Some of these immune functions including defense are, in fact, carried out by non-immune cells, including microbes, thus leading to the “co-immunity” (Chiu et al. 2017) of a host together with its microbiota. Additionally, we submit that other issues which have so far received little attention from philosophers require consideration.

First, it should be noted that, arguably, most immunologists and physicians think of defense narrowly in terms of resistance, i.e., the clearance of pathogens via destruction, expulsion, or containment. However, an organism also relies on another defense strategy called ‘disease tolerance’ (Schneider and Ayres 2008). While the resistance strategy is defined by reducing the pathogen burden, the consequence of which is always some degree of immunopathology, the tolerance strategy amounts to reducing the negative impact of pathogen-induced damage and immunopathology by decreasing the susceptibility of the host to tissue damage. In other words, disease tolerance influences survival without affecting (pathogen) burden. Importantly, disease tolerance cannot be reduced to a strategy of defense from ‘pathogenic non-self’ since it plays a role in a variety of other conditions such as sterile inflammation, obesity etc. It is also realized by components that include, but also are not exhausted by, immune components. Thus, disease tolerance suggests that defense requires the involvement of multiple physiological systems (Schneider 2021a; Zach and Greslehner 2022).

Second, there is an important consequence for researchers from other fields who want or have to include the immune system in their own considerations. One example is neuroimmunology, where the multi-faceted interactions and cross-talk between the nervous and immune system are studied. Thinking that the immune system is only about defense against pathogens and disease, the nervous system only about cognition and behavior would be—if not a fallacy—at least a misconception and missed opportunity to see adequately how these systems are connected and operate. The focus on defense and disease has biased the field for most of its (brief) history towards a narrow set of phenomena. While the role of the immune system is important for understanding several neurological diseases (e.g., multiple sclerosis and dementia), and likewise the nervous system is important in immunological diseases, thinking in terms of defense does these overlapping domains a big disservice. Many other non-disease-related phenomena such as cognition in non-pathological contexts (Steinman 2004; Marin and Kipnis 2013) are largely influenced by the interplay of both systems, which cannot be properly accounted for when thinking in terms of diseases and defense. This very lesson is equally important for other areas of research where immunology is frequently introduced with defense in mind. In fact, the important roles of the immune system in non-defensive interactions with other systems like the nervous system, endocrine system, the microbiota, organismal development etc. all show that the immune system is not just a defense unit against pathogens.

### The bottom line

In summary, we argue that to acquire a general understanding of immunity, i.e., what the immune system is, what it does and why it sometimes fails, the idea that the immune system is first and foremost a defense system characterized in terms of strength or weakness must be reconsidered.

We think that our discussion so far shows that much needed nuance is lost when relying on the notions of strength and weakness. It is worth noting that one can always develop a liberal enough interpretation of the terms such as ‘defense’ and ‘strength’ so that it fits *any* and *all* descriptions of observed phenomena. This would surely be motivated by the wide-spread and deeply rooted mindset which relies on such terminology. In most cases, however, such an interpretation would always be more or less far-fetched, and it would come at the cost of equivocating otherwise distinct features of the immune system, ultimately resulting in notions devoid of any precise meaning.

## Rethinking immunity

In this section we want to turn the page by introducing a positive account of immunity, that is, a framework that is better suited for understanding what the immune system is, what it does, and why it sometimes fails. Such a framework consists of three key concepts—contextuality, regulation, and trade-offs (CORETO)—that together best account for the nature of the immune system and the immune response, or so we argue. By *contextuality* we mean the fact that the outcome of an immune response is essentially dependent on the context which in turn is determined by a multitude of factors, including the kinetics of the response. Any characterization of immunity that omits the contextual nature is thus doomed to misrepresent what is going on. Similarly, *regulation* takes center stage. During the course of an (e.g., acute inflammatory) immune response, the various mediators involved trigger a cascade of events leading to a build-up of molecules and cells in various tissues which further amplify the response by recruiting more and more immune mediators. As is the case with any such cascade, however, there must be a way to keep it from spiraling out of control. A vast array of feedback mechanisms serves that very purpose. In other words, any immune function must be finely tuned and tightly regulated by both internal and external signals. Finally, the immune system exhibits various *trade-offs*: having a particular feature very often confers some benefit to the host with respect to some particular condition, while at the same time that very same feature also puts the host in disadvantage with respect to another condition. Taking these three key concepts into account, the simplistic idea that immunity or the immune response can be fruitfully conceived of in terms of ‘strong’ or ‘weak’ defense is flawed.

The concepts of contextuality, regulation, and trade-offs are anything but new to immunologists, as each of them commonly features in published work. However, these concepts usually appear as descriptors of discovered states of affairs. What is missing so far is an explicit and systematic discussion of these concepts that would highlight their usefulness for understanding general organizing principles of immunity—and accommodate a wider range of immune phenomena other than ‘strong defense’. The purpose of this section is to fill this gap.

### Contextuality

Rather than the immune system being activated only occasionally when facing threats, the immune system is in fact constantly active in maintaining various functions and interacting with its (internal and external) environment,^6^ with the outcome of these interactions being context-dependent through and through. Furthermore, it is important to point out that such contextuality comes in many layers.

#### General immune functions: levels and players

On the most general level, the contextuality of an immune response concerns the particular function at play, whether that be defense, tissue repair, the maintenance of homeostasis, the clearance of debris, including senescent cells, or a role in development. Which of these functions is triggered depends on the particular situation, driven by the integration of various signals and immune mediators such as cytokines.

One and the same thing can—and often does—fulfill different general functions, depending on what is going on. For instance, IFN-β, a type I interferon (IFN-I), is produced by epithelial cells and specialized subsets of immune cells early in a response to a viral infection. The infected cells start producing IFN-Is, the actions of which then interfere with viral replication in many ways. In addition to its presence in increased concentration during infection, IFN-β is also constitutively expressed at low levels and contributes to tissue homeostasis (Stefan et al. 2020). Similarly, many immune cells enact various general functions ranging from inflammatory responses to tissue repair and remodeling, depending on the context, exhibiting the phenomenon of cellular plasticity (Laurent et al. 2017).

Contextuality also pertains to the specifics of a given immune function. Recall disease tolerance—far from being a strict matter of an either-or strategy, resistance (the clearance of pathogens) and tolerance may be located on a spectrum and, moreover, are pathogen-specific (or more generally, condition-specific). For example, infection-induced anorexia, a kind of sickness behavior associated with infection, increases the tolerance to infection by *Salmonella typhimurium* while it decreases resistance to infection by *Listeria monocytogenes* in *Drosophila melanogaster* (Ayres and Schneider 2009). Morbidity and mortality in an infection may be due to a failure in resistance. However, if a comparable pathogen burden is found in hosts with different morbidity or mortality profiles despite the evidence of effective resistance, the pathology may result from a failure in tolerance (Medzhitov et al. 2012). To give a recent example, think of COVID-19 which affects infected individuals very differently. Some studies have shown that there may be no significant difference in viral load in symptomatic versus asymptomatic cases of infections with SARS-CoV-2 (Lee et al. 2020), meaning that the course of disease may, at least to some degree, reflect individual differences in susceptibility to tissue damage: the problem may be one of disease tolerance, i.e., reducing the tissue damage due to SARS-CoV-2 and immunopathology, rather than resistance, i.e., clearance of SARS-CoV-2 (Ayres 2020).^7^

#### Contextualizing microbes

Notwithstanding the fact that some microbes seem purely pathogenic in a given species such as humans, a large number of microbes are pathogenic only under certain conditions. In fact, pathogenicity is a complex and dynamic relation between the host and the microbe (Méthot and Alizon 2014). Only within the last few decades has the importance of the microbiome started to be fully appreciated in health and disease (Turnbaugh et al. 2007), offering additional ‘holobiont’ or ‘superorganism’ perspectives in immunology (Eberl 2010).

Many viruses also exhibit interesting contextual features, even though they have been predominantly associated with purely pathogenic or otherwise detrimental effects on the host. When we think of viruses, one immediately thinks of pathogens causing diseases which scourge humanity. However, viruses are also an oft-neglected key part of the microbiome, ignored for a number of reasons which include not only methodological difficulties but also our biased perception owing to biomedical microbiology mostly being driven by a desire to understand pathogens. They not only play a role as pathogens; in the study of good health, the human virome is also a central factor which has until recently remained largely unexplored, remaining viral ‘dark matter’ (Liang and Bushman 2021). Pradeu (2016a) provides an intriguing overview, showing that while many viruses have become indispensable to host development, others confer protection against disease: for example, although many of the herpesviruses put individuals at risk of developing diseases, in their latent form several of the herpesviruses also provide protection against some bacterial infections such as by *Listeria monocytogenes* or *Yersinia pestis* (Barton et al. 2007).

#### Contextualizing the immune system

Another ‘contextuality layer’ concerns the nature of the immune system itself. The immune system, far from being monolithic, consists of a vast network of interacting parts that can be carved up in multiple ways, but none is clear-cut.

One’s immune status is often characterized as being in an immunocompetent or immunocompromised state, but both characterizations also exhibit contextual features. Immunocompetence is not something ‘static’ or ‘given’ because it evolves over time, most notably during development and aging. Intriguingly, the temporal changes in the workings of the innate and adaptive immune system, with consequences for its functions, also relate to circadian rhythms, i.e., they oscillate between day and night regimes (Keller et al. 2009; Druzd et al. 2017). Moreover, the different outcomes of an immune response across individuals also result from various polymorphisms such as in the human leukocyte antigen (HLA) loci; these polymorphisms are not defects. Finally, the susceptibility of an individual is also heavily influenced by the individual’s immune history^8^: a prior infection affects immunity and pathology to the next infection (Hussell 2016) or to a tissue transplant (Sachs 2003). For example, cross-reactive memory T cells can have a protective effect, but they can also lead to immunopathology (Welsh and Selin 2002). Such contextual features do not concern merely the adaptive arm, but extend to the innate immunity as well as tissue-specific microenvironment: the altered state of lung tissue due to an infection influences the outcome of a later infection, with some sequences of certain infections having beneficial effect on a later response, while other sequences exhibiting harmful effects (Hussell 2016).

Yet immunocompromised individuals are characterized by the presence of some sort of immunodeficiency or by being in an immunosuppressed state. Since immunosuppression will be brought up in the next section, we shall leave discussion until then. Immunocompromised individuals have been intuitively considered as individuals with ‘weak immunity’. However, closer inspection will reveal substantial problems with such an intuition.

With respect to immunodeficiency, it is customary to distinguish between primary or inherited immunodeficiency which are due to genetic defects (e.g., IPEX discussed above), and secondary or acquired immunodeficiency, which are quite common and which may arise from various causes such as due to infection (e.g., HIV), surgery (e.g., splenectomy, neonatal thymectomy), or malnutrition. It is important to note that immunodeficient people cannot be, in general, thought of as individuals who possess ‘weaker’ immune system. Rather, the discussion on immunodeficiency illustrates yet again the contextual nature. First of all, it is wrong to think that an immunodeficiency necessarily confers a system-wide defect. It is true that some defects (such as IPEX) prove fatal and others, such as a variety of severe combined immunodeficiencies (SCID), leave the host extremely susceptible to a wide range of conditions. However, other defects make the host overly susceptible only to specific infections (e.g., due to splenectomy), while some may not even clinically manifest themselves (e.g., thymectomy in adulthood). Some immunodeficiencies, such as the relatively common deficiency of IgA production, does not leave most of the individuals overly susceptible to infection, possibly owing to the compensation by IgM secretion (Yel 2010). A specific form of malnutrition providing protection in malaria infection—an example noted above—further illustrates the contextual nature of immunodeficiencies.

The fact that a great many immunodeficiencies do not manifest themselves in a clinically relevant manner may be explained by the fact that the immune system, as with many other biological systems, is adaptive and notorious for exhibiting biological redundancy, meaning that, in many instances, should one pathway or ‘player’ fail, another may step in and take over. Such redundancy thus gives rise to the phenomenon of robustness, that is, that the immune system is, on average, capable of functioning adequately even if some parts exhibit certain defects.^9^

### Regulation

Regulation takes place on multiple levels of organization. The complement system pathways are regulated by the presence of a set of proteases in the plasma or molecules constitutively expressed on cell surfaces. Co-stimulatory molecules provide an additional check on the activation of many types of immune cells by ensuring that an immune response is triggered in an appropriate context. Indeed, possibly unwanted responses are prevented by these mechanisms. The proper trafficking pattern is regulated by specific adhesion molecules and chemokines and their receptors. Cytokines are another major player in the regulatory processes as they influence cell responsiveness, proliferation, and differentiation. A specialized subset of regulatory T cells is necessary for the correct functioning of the immune system. In fact, recent studies have shown that many, if not all, types of immune cells exhibit both an effector and a regulatory phenotype (see, e.g., Mantovani et al. 2011 for a discussion on neutrophils). Malfunction of the immune regulatory mechanisms—immune dysregulation—is at the heart of many pathologies. For instance, “the severe acute respiratory syndrome coronavirus 2 (SARS-CoV-2) pandemic has reminded us of the critical role of an effective host immune response and the devastating effect of immune dysregulation” (Fajgenbaum and June 2020, p. 2255).

#### Everything in moderation and in context

The crucial regulatory aspect of the immune response suggests that a physiologically adequate response must be neither ‘too strong’, nor ‘too weak’, but just about right (Sakaguchi 2006). What ‘right’ means here depends not only on the proper regulation of the response but also on the specific kind of response which must be adjusted to the particular task, i.e., the contextual nature of what is going on. A response that is too vigorous results in much tissue damage. A response that is too permissive may not clear the pathogen, which may then establish a chronic infection.

While some dysregulation may be transient, e.g., due to a lack of those nutrients required primarily to maintain the function of a specific cell population (secondary immunodeficiency), dysregulation may also be persistent, such as on account of a mutation in a molecular regulator of inflammation which gives rise to autoinflammatory diseases characteristic of inflammation even in the absence of infection.

Some forms of dysregulation may have recurrent clinical manifestation, whereas some others can remain hidden until showing in a particular clinical context. For example, some of the patients developing severe COVID-19 harbor antibodies against some subsets of their own IFNs (Bastard et al. 2020). As discussed above, IFN-Is are important early in an anti-viral response. However, the presence of autoantibodies against IFNs found predominantly in subsets of male patients with severe COVID-19 leads to the limited availability of IFNs and results in a delayed response and an improper recruitment of other immune cells. Thus, the autoantibodies against IFN-Is contribute to an imbalanced host response. Furthermore, a greater variety of other autoantibodies against immunomodulatory proteins have been found in patients with COVID-19 compared to uninfected controls, with the analysis suggesting the existence of both pre-existing and newly induced autoantibodies following the infection (Wang et al. 2020).

Similarly, it is well established that the elderly exhibit low-grade chronic inflammation, which, although it does not appear to cause clinical problems, does contribute to disease. In general, the very young as well as the old are more susceptible to infections. However, this simple fact is not meaningfully captured by the concept of a ‘weaker immunity’. Rather, the aging organism exhibits an immunosenescent phenotype of the innate immune system, characteristic of the condition of inflammaging which contributes to dysregulation by creating a constitutive pro-inflammatory environment (Shaw et al. 2010). As a result, some of the responses of the aging organism are improperly *enhanced*—hence dysregulated—which, together with other age-related changes such as the shift in the relative numbers of some immune cell subsets and their phenotypes may, at least in part, explain why age is a major risk factor in COVID-19 (Brodin 2021; Schultze and Aschenbrenner 2021).^10^

#### Regulation beyond defense

The importance of the regulatory processes can also be illustrated with reference to the maintenance of homeostasis, tissue repair, and disease tolerance. Mice with impaired Toll-like receptor signaling cannot control homeostasis (or the development and maturation) of intestinal epithelium; such failure leads to chronic inflammation of the gut and the associated tissue damage as seen in inflammatory bowel diseases (Rakoff-Nahoum et al. 2004). Regulatory T cells have been shown to play an important role in promoting muscle repair and reducing inflammation upon injury in mice; depleting these cells leads to a disorganized tissue structure (Burzyn et al. 2013; Truchetet and Pradeu 2018). Similarly, just as a dysregulated immune response leads to pathology, disease tolerance must also be controlled if a pathology is to be avoided, e.g., fibrosis resulting from an excessive—dysregulated—tissue repair (Medzhitov et al. 2012).

#### Immune modulation

Although the immune system appears sophisticated and effective, it nevertheless does not always get things ‘right’ (from our human perspective), needing to be nudged in the right direction. Drugs with immunomodulatory effects serve precisely this purpose. Rather than accounting for immune modulation in terms of making a response stronger or weaker, the way to adequately capture what is going on is to think in terms of specifically intervening and triggering, constricting, or facilitating certain responses.

As we saw, part of the problem in some severe cases of COVID-19 may be a delayed anti-viral response due to the presence of autoantibodies against certain subsets of IFNs. Some researchers are testing the administering of exogeneous IFN-β (Bastard et al. 2021), the motivation being that these IFNs may enhance the immune response in non-specific ways. In fact, IFN-β is used in the treatment of diseases of viral origin (Guarda et al. 2011). However, IFN-β has also been found to reduce a response rather than to enhance it, and as such it has proven useful in treating patients with multiple sclerosis, owing to its effect on the reduction of IL-1 production, thereby limiting a powerful mediator of inflammation (Guarda et al. 2011).

The immune system also often fails to respond against purified proteins. Rather, because such proteins are usually poorly immunogenic, they often induce a state of immunological tolerance. In many contexts this is usually beneficial. However, it presents an obstacle in the design of vaccines based on the use of purified proteins, including vaccines based on the toxoid design like the tetanus toxoid, or the subunit vaccines. To overcome these difficulties, scientists have developed a number of adjuvants—substances that increase reactivity, most notably by stimulating innate sensor pathways. Although many adjuvants are routinely used in experimental research, only a few are approved for clinical use in humans. The problem is that most adjuvants cause dangerously excessive inflammation. Thus, ‘boosting’ an immune response must always be kept within strict limits.

Consider also the case of disease tolerance. As Medzhitov et al. (2012) argue, ‘boosting’ an immune response when the problem is a failure of tolerance may prove ineffective or even detrimental, whereas ‘boosting’ tolerance may provide health benefits by limiting tissue damage caused either by the pathogen directly or by the immune response to the microbe. However, here again it is important to stress that any such action must be carefully regulated. Although some tolerance mechanisms appear to be at work at the basal level, others are inducible and work at the expense of normal tissue function. Thus, much like the mechanisms of resistance, tolerance also comes at a cost. Furthermore, tolerance mechanisms also require tight control; otherwise, they result in pathology, as illustrated by the above example of fibrosis.

In other cases, it is desirable to ‘attenuate’ an immune response rather than ‘boost’ it. Transplant patients take immunosuppressive drugs in order for the transplanted organ not be rejected.

Cancer chemotherapy provides another interesting example. While it is true that chemotherapy is generally associated with an increased risk of infection due to its immunosuppressive effect and as such it is often categorized as a secondary immunodeficiency, it has also been found that certain chemotherapeutic drugs such as anthracyclines work, in part, by increasing the immunogenicity of the tumor cells, thus increasing the anti-tumor immune responses (Alizadeh and Larmonier 2014). Consequently, chemotherapy may be said to result in general suppression by lowering the count of immune cells, while at the same time it may ‘boost’ a specific immune response, provided that enough antigen-specific T cells survive the therapy.

It should also be noted that the idea of modulation presupposes that there is something to be modulated. In the above cases, this assumption was implicit. Given that the immune system is not monolithic, but rather consists of a large number of diverse molecules, cell populations, and several kinds of specialized organs, it may not always be the case that there is something to be modulated. Indeed, consider again the removal of the thymus at an early age which prevents the development of mature T cells: there is nothing left to modulate.

### Trade-offs

Given the complexity of the biological systems and the discussion in the two previous sections, it should come as no surprise that the workings of the immune system exhibit numerous trade-offs on multiple levels of organization. This means that something is (at least partly) incompatible with something else; put another way, it is not possible to have both at the same time.

#### Defense

It is important to realize that there is a trade-off between resisting an infection, i.e., the clearance of pathogens, and the tissue damage arising from the immune response, i.e., the immunopathology. A ‘strong’ response, in this sense, may be associated with the vigorous clearance of pathogens while giving rise to a cytokine storm, an immune-mediated life-threatening condition.^11^ Yet another important kind of trade-off is made with respect to evolutionary fitness. The host has to find a balanced immune response and allocate the resources and energy, as any immune response comes at a cost (Lochmiller and Deerenberg 2000). On the flip-side of this coin we find the trade-offs with respect to parasite virulence (Alizon et al. 2009).

Medzhitov et al. (2012) note that a response against microbe ‘A’ can be incompatible with tolerating microbe ‘B’, giving rise to the phenomenon of negative preconditioning. They also note that coinfection, e.g., a viral infection of the respiratory tract followed by a respiratory bacterial infection, often results in severe morbidity and mortality which is usually thought to be the consequence of compromised immunity. Indeed, Eberl (2016) proposes that the cross-regulation of types of responses may be the problem,^12^ i.e., viral infection induces a type 1 response which inhibits the type 3 responses crucial for clearing extracellular bacterial infections. However, as Medzhitov et al. suggest, it is also possible that the inducible tolerance to the particular viral infection is incompatible with tolerance to the respiratory bacterial infection which may be why that kind of coinfection is dangerous.

Similarly, as noted above, while some disease tolerance mechanisms are constitutive, others are inducible and come at a cost: they work at the expense of normal tissue function. Thus, there is a trade-off between normal tissue function and an increased tolerance to tissue damage.

The idea that one’s particular genotype can influence the susceptibility of an individual is also well established. However, the same genes that confer protection can also make the individual susceptible to other conditions. For example, using genome-wide association studies, the genetic variation in human leukocyte antigen (HLA) molecules has been established as one of the strongest predictors of HIV-1 control (Pereyra et al. 2010). While the HLA-B*27 allele, by virtue of its mechanistic function, has been found to increase resistance to HIV-1, it also leaves the host at greatly increased risk of developing ankylosing spondylitis, an autoimmune disease (Murphy and Weaver 2017).

#### Beyond defense

Turning to functions other than defense, there are trade-offs between a beneficial function under some conditions and a detrimental effect under other conditions. For instance, cellular plasticity of macrophages is crucial in the process of tissue repair but its presence in cancer allows for the occurrence of pro-tumoral effects (see Truchetet and Pradeu 2018). Similarly, following liver damage, the transient induction and accumulation of senescent cells help to resolve fibrosis (Krizhanovsky et al. 2008). However, senescent cells need to be cleared by the immune system since their prolonged existence is considered detrimental. In the aging organism such detrimental effects become apparent as these cells accumulate, owing either to a decrease in the clearance capabilities of the immune system or to an increase in the generation of such cells which exceeds the capacity of the immune system to clear them, or to some combination of the two (Rodier and Campisi 2011).

#### Immune modulation

Various trade-offs also arise when therapeutically manipulating the immune responses. Recall the use of ipilimumab in cancer treatment and its known side effect—the onset of severe autoimmunity in some cases. To give another example, in order to avoid transplant rejection, patients receiving transplants are put on non-specific immunosuppressive drugs for life, which, however, leave them more susceptible to infection and cancer.

## Conclusion

Discussions of the immune system are often riddled with phrases such as ‘strong/weak immunity and response’. In fact, this is part and parcel of immunological writings, owing to the long tradition of viewing the immune system as a defense system and the intuitive grasp of the notions of ‘strength’ and ‘weakness’.

Metaphors play a significant—and unavoidable—role in shaping the narrative and perspective on the immune system as a strong/weak defense system. While they serve as tools for understanding, navigating, and organizing what would otherwise constitute a complex mess of individual facts, they may also shape a narrow perspective, overlooking other important aspects, as we have demonstrated in this paper.

We have pointed out that the immune system is involved in functions other than defense, and that the function of defense is also realized by host components that are traditionally not regarded as part of the host immune system. Furthermore, even with defense in mind, there are other strategies than just the elimination of pathogens, as showcased by disease tolerance. Finally, interactions between the immune and other systems are not restricted to defense and pathologies.

With this in mind, we examined several interpretations of the notions of ‘strong/weak immunity and response’. One prominent usage in immunological literature draws on the use of quantitative immunological assays. We argued that interpreting such measurements in terms of the strong/weak framework does little to improve the quantitative statements. On the normative reading of these notions, one may associate strength with positive—and weakness with negative—connotations, respectively. However, such a picture turns out to be misleading, as ‘strong’ immunity or response is not necessarily desirable, and likewise ‘weak’ immunity is not always detrimental. Paradoxical connotation stems from the fact that an immune condition can oftentimes be viewed as both ‘strong’ and ‘weak’, and preferring one over the other is rather arbitrary. Given that the immune system is not monolithic, many particular functions may not be amenable to change, whereby the intuitive idea of making the immune system stronger or weaker breaks down. Moreover, many immunological phenomena and functions cannot be meaningfully captured by these notions. Finally, the strong/weak framework mischaracterizes the nature of the interactions between the immune system and other physiological systems, and what their respective contributions are. Taken together, we argued that while the strong/weak framework might seem intuitively appealing, it turns out that it is not well defined but rather ill-suited and can be misleading. Furthermore, our systematic analysis suggests that much nuance is lost when relying on the notions of strength and weakness. Although one can always develop a liberal enough interpretation of the terms such as ‘defense’ and ‘strength’ so that it fits *any* and *all* descriptions of observed phenomena, such a loose interpretation would render the notions devoid of meaning. Thus, we argued that the use of the strong/weak framework should be reconsidered.

Rather than merely providing a critique, we also proposed an alternative, positive framework that does justice to what is known about how the immune system operates in both health and disease. Our framework consists of three key concepts: contextuality, regulation, and trade-offs (CORETO). All immune-related phenomena require a contextual understanding; otherwise, one would fail to understand why a phenomenon may appear desirable in one context and detrimental in another. Regulation plays a paramount role in accounting for many ways in which the immune system operates or dysfunctions. Finally, one and the same component of the immune system that confers a particular benefit is also responsible for a poor outcome regarding another condition. Thus, the immune system exhibits numerous trade-offs. Although these concepts can be found in immunological literature, an explicit and systematic consideration had been missing. In this paper we provided such an explicit analysis and proposed to unify these concepts into a single framework. We proposed our framework in order to gain a better understanding of the organizing principles of immunity that will allow us to address the role of the immune system in health and disease.

## Data Availability

Not applicable.

## Footnotes

1 In fact, immunology has been investigated by philosophers in relation to a great many topics: the self/non-self theory of immunogenicity (Tauber 1994; Pradeu 2012), biological individuality and the related holobiont and ecological views (Pradeu 2016b; Schneider 2021b), the use of metaphors in biological, including immunological thinking (Martin 1994; Tauber 1994), and a variety of more specific notions such as immunological balance (Swiatczak 2013).

2 The original meaning of ‘immunity’ derives from Latin ‘exempt from service’. The notion of immunity and other related concepts have been a subject of philosophical analysis before (see, e.g., Fleck 1979; Tauber 2017). Roughly, ‘immunity’ is sometimes used to capture the general immune status of an individual. Other times, ‘immunity’ picks out a specific target, such as immunity to a particular pathogen. However, this does not exhaust the many possible meanings; for instance, the notion of immunity also applies to the naïve immune status, or one may have in mind specific notions such as protective or sterilizing immunity. Carefully disentangling the many meanings of ‘immunity’ in their different contexts is a Herculean task and beyond the scope of this paper.

3 Of course, quantitative measurements are crucial in a variety of contexts such as diagnosis, prognosis, treatment, etc. However, very little if anything at all is gained by assigning ‘weakness’ or ‘strength’ to them.

4 Of course, it is not guaranteed that an individual develops long-lasting immune memory as a result of an infection or inoculation. Even if successful, sterilizing immunity which would provide full protection against infection is not guaranteed.

5 One often encounters the distinction between an immunocompetent and an immunocompromised individual, the latter meaning either an individual with an immunodeficiency, or an individual on immunosuppressive drugs. Interestingly, providing a definition of an immunocompetent individual proves difficult and is rarely the subject of debate. Instead, an implicit reliance upon a *negative* definition—an individual who exhibits no (known) deficiency or is not immunosuppressed—seems to be the rule.

6 This phenomenon is perhaps most vividly illustrated by those mucosal surfaces which constantly interact with the microbiome, but it also holds true for systemic immunity (Eberl 2016).

7 Similarly, Medzhitov et al. (2012) have proposed that the concept of tolerance may apply to phenomena such as the “Typhoid Mary”, i.e., cases where a carrier remains healthy (asymptomatic) perhaps due to having a high level of tolerance to the particular pathogen.

8 This is also why even two genetically identical twins are immunologically different from one another.

9 What it means for a system to be robust, and what it means for it to exhibit redundancy, are complex questions since these concepts are applied to a wide range of phenomena. For instance, drawing on Kitano (2004), Truchetet and Pradeu (2018) define robustness as the maintenance of specific functionalities of a given system against internal and external perturbations. Truchetet and Pradeu also analyze robustness in pathological conditions, distinguishing robustness as dysfunction, when robustness is ‘hijacked’ in conditions such as AIDS and some types of cancer, and dysfunctional robustness, when a system should be robust but is not, in cases such as tissue repair.

10 Interestingly, evidence of the sustained immune dysregulation of several cell subsets has been found in both hospitalized and non-hospitalized infected individuals. Moreover, some of these changes were found to increase over time in non-hospitalized patients. The lasting effects on subsequent infections or inflammatory diseases are yet to be determined (Files et al. 2021).

11 Some authors argue that the trade-off between resistance and immunopathology can be resolved, to some extent, by tolerance mechanisms. Since these limit tissue damage, they allow for a prolonged duration of the immune response (Martins et al. 2019).

12 Several cues suggest that an improper cross-regulation—combined with certain kinetics of the responses—may also arise in some cases of COVID-19, where an increase in type 2 effectors has been observed in severe COVID-19, in contrast to a burst of type 1 and type 3 responses followed by their subsequent progressive reduction in moderate COVID-19 (Lucas et al. 2020b).
